# Use and impact of high intensity treatments in patients with traumatic brain injury across Europe: a CENTER-TBI analysis

**DOI:** 10.1186/s13054-020-03370-y

**Published:** 2021-02-23

**Authors:** Jilske A. Huijben, Abhishek Dixit, Nino Stocchetti, Andrew I. R. Maas, Hester F. Lingsma, Mathieu van der Jagt, David Nelson, Giuseppe Citerio, Lindsay Wilson, David K. Menon, Ari Ercole, the CENTER-TBI investigators and participants, Andrew I. R. Maas, Nino Stocchetti, Hester F. Lingsma, Giuseppe Citerio, David K. Menon, Ari Ercole, Cecilia Åkerlund, Krisztina Amrein, Nada Andelic, Lasse Andreassen, Gérard Audibert, Philippe Azouvi, Maria Luisa Azzolini, Ronald Bartels, Ronny Beer, Bo-Michael Bellander, Habib Benali, Maurizio Berardino, Luigi Beretta, Erta Beqiri, Morten Blaabjerg, Stine Borgen Lund, Camilla Brorsson, Andras Buki, Manuel Cabeleira, Alessio Caccioppola, Emiliana Calappi, Maria Rosa Calvi, Peter Cameron, Guillermo Carbayo Lozano, Ana M. Castaño León, Simona Cavallo, Giorgio Chevallard, Arturo Chieregato, Mark Coburn, Jonathan Coles, Jamie D. Cooper, Marta Correia, Endre Czeiter, Marek Czosnyka, Claire Dahyot-Fizelier, Paul Dark, Véronique De Keyser, Vincent Degos, Francesco Della Corte, Hugo den Boogert, Bart Depreitere, Dula Dilvesi, Abhishek Dixit, Jens Dreier, Guy-Loup Dulière, Erzsébet Ezer, Martin Fabricius, Kelly Foks, Shirin Frisvold, Alex Furmanov, Damien Galanaud, Pedro A. Gomez, Francesca Grossi, Deepak Gupta, Iain Haitsma, Eirik Helseth, Peter J. Hutchinson, Stefan Jankowski, Faye Johnson, Mladen Karan, Angelos G. Kolias, Daniel Kondziella, Evgenios Koraropoulos, Lars Owe Koskinen, Noémi Kovács, Ana Kowark, Alfonso Lagares, Steven Laureys, Didier Ledoux, Aurelie Lejeune, Roger Lightfoot, Alex Manara, Costanza Martino, Hugues Maréchal, Julia Mattern, Catherine McMahon, Tomas Menovsky, Benoit Misset, Visakh Muraleedharan, Lynnette Murray, Ancuta Negru, Virginia Newcombe, József Nyirádi, Fabrizio Ortolano, Jean François Payen, Vincent Perlbarg, Paolo Persona, Anna Piippo-Karjalainen, Horia Ples, Inigo Pomposo, Jussi P. Posti, Louis Puybasset, Andreea Radoi, Arminas Ragauskas, Rahul Raj, Jonathan Rhodes, Sophie Richter, Saulius Rocka, Cecilie Roe, Olav Roise, Jeffrey V. Rosenfeld, Christina Rosenlund, Guy Rosenthal, Rolf Rossaint, Sandra Rossi, Juan Sahuquillo, Oddrun Sandrød, Oliver Sakowitz, Renan Sanchez-Porras, Kari Schirmer-Mikalsen, Rico Frederik Schou, Peter Smielewski, Abayomi Sorinola, Emmanuel Stamatakis, Nina Sundström, Riikka Takala, Viktória Tamás, Tomas Tamosuitis, Olli Tenovuo, Matt Thomas, Dick Tibboel, Christos Tolias, Tony Trapani, Cristina Maria Tudora, Peter Vajkoczy, Shirley Vallance, Egils Valeinis, Zoltán Vámos, Gregory Van der Steen, Roel P. J. van Wijk, Alessia Vargiolu, Emmanuel Vega, Anne Vik, Rimantas Vilcinis, Victor Volovici, Petar Vulekovic, Guy Williams, Stefan Winzeck, Stefan Wolf, Alexander Younsi, Frederick A. Zeiler, Agate Ziverte Hans Clusmann, Daphne Voormolen, Jeroen T. J. M. van Dijck, Thomas A. van Essen

**Affiliations:** 1 Center for Medical Decision Sciences, Department of Public Health, Erasmus MC– University Medical Center Rotterdam, Rotterdam, The Netherlands; 2 Division of Anaesthesia, University of Cambridge, Addenbrooke’s Hospital, Cambridge, UK; 3 Department of Pathophysiology and Transplants, University of Milan, Milan, Italy; 4 Fondazione IRCCS Ca’Granda, Ospedale Maggiore Policlinico, Milan, Italy; 5 Department of Neurosurgery, Antwerp University Hospital and University of Antwerp, Edegem, Belgium; 6 Department of Intensive Care Adults, Erasmus MC– University Medical Center Rotterdam, Rotterdam, The Netherlands; 7 Section for Perioperative Medicine and Intensive Care, Department of Physiology and Pharmacology, Karolinska Institutet, Stockholm, Sweden; 8 School of Medicine and Surgery, University of Milan-Bicocca, Milan, Italy; 9 Neurointensive Care, San Gerardo Hospital, ASST-Monza, Monza, Italy; 10 Division of Psychology, University of Stirling, Stirling, UK

**Keywords:** Therapy intensity level, Barbiturates, Hypothermia, Hyperventilation, Decompressive craniectomy, Traumatic brain injury

## Abstract

**Purpose:**

To study variation in, and clinical impact of high Therapy Intensity Level (TIL) treatments for elevated intracranial pressure (ICP) in patients with traumatic brain injury (TBI) across European Intensive Care Units (ICUs).

**Methods:**

We studied high TIL treatments (metabolic suppression, hypothermia (< 35 °C), intensive hyperventilation (PaCO_2_ < 4 kPa), and secondary decompressive craniectomy) in patients receiving ICP monitoring in the ICU stratum of the CENTER-TBI study. A random effect logistic regression model was used to determine between-centre variation in their use. A propensity score-matched model was used to study the impact on outcome (6-months Glasgow Outcome Score-extended (GOSE)), whilst adjusting for case-mix severity, signs of brain herniation on imaging, and ICP.

**Results:**

313 of 758 patients from 52 European centres (41%) received at least one high TIL treatment with significant variation between centres (median odds ratio = 2.26). Patients often transiently received high TIL therapies without escalation from lower tier treatments. 38% of patients with high TIL treatment had favourable outcomes (GOSE ≥ 5). The use of high TIL treatment was not significantly associated with worse outcome (285 matched pairs, OR 1.4, 95% CI [1.0–2.0]). However, a sensitivity analysis excluding high TIL treatments at day 1 or use of metabolic suppression at any day did reveal a statistically significant association with worse outcome.

**Conclusion:**

Substantial between-centre variation in use of high TIL treatments for TBI was found and treatment escalation to higher TIL treatments were often not preceded by more conventional lower TIL treatments. The significant association between high TIL treatments after day 1 and worse outcomes may reflect aggressive use or unmeasured confounders or inappropriate escalation strategies.

**Take home message:**

Substantial variation was found in the use of highly intensive ICP-lowering treatments across European ICUs and a stepwise escalation strategy from lower to higher intensity level therapy is often lacking. Further research is necessary to study the impact of high therapy intensity treatments.

***Trial registration*:**

The core study was registered with ClinicalTrials.gov, number NCT02210221, registered 08/06/2014, https://clinicaltrials.gov/ct2/show/NCT02210221?id=NCT02210221&draw=1&rank=1 and with Resource Identification Portal (RRID: SCR_015582).

## Background

Limiting the impact of secondary insults by controlling harmful levels of intracranial pressure (ICP) is an essential part of Traumatic Brain Injury (TBI) care in the intensive care unit (ICU). Interventions used to reduce ICP are typically titrated to balance their clinical effect against their side effects, which may be significant or even life-threatening. The intensity of such interventions can be quantified by the therapy intensity level (TIL) score. The TIL score was first introduced in 1987 [1], and has been revised over the years into a more advanced scoring system [2] which was recently validated [3]. Conceptually, the stepwise approach to treatment of raised ICP aims to use low tier therapies in the first instance, reserving more aggressive (and hazardous) high TIL treatments only for when these fail.

Despite this proposed framework for rational use of ICP therapies, past studies have found wide variations between centres in their deployment [4, 5]. Some of this variation may reflect either therapeutic nihilism or inappropriately aggressive use (as high intensity treatment can be clinically burdensome and consumes more ICU resources). While some studies report efficacy of high TIL therapies when properly targeted in terms of patient group and timing [6], other publications have given rise to concern that they may be ineffective in improving ultimate outcomes, and result in increased survival with severe disability [7, 8].

Therefore, the aim of this study is to investigate the variation in the use of high TIL therapies in clinical practice and explore the impact on clinical outcome in patients with TBI in European ICUs.

## Methods

### CENTER-TBI study/ study population

Data from the Collaborative European NeuroTrauma Effectiveness Research in Traumatic Brain Injury (CENTER-TBI) study were used for this analysis (clinicaltrials.gov NCT02210221). CENTER-TBI recruited patients with TBI, presenting between December 19, 2014 and December 17, 2017 [9, 10]. Inclusion criteria for the CENTER-TBI study were: A clinical diagnosis of TBI, an indication for brain computer tomography (CT) and presentation within 24 h post-injury. Patients with severe pre-existing neurological disorders were excluded. For this study we selected patients of 14 years and older admitted to the ICU with documented daily measurements on the TIL scale for the first 7 days since admission to the ICU and with ICP monitoring.

### Therapy intensity level

In the CENTER-TBI study, the most recent TIL scale is used [3] which measures and quantifies the intensity of ICP lowering treatments (and includes common data elements harmonized with the paediatric TIL scale [2]). The TIL scale consists of 8 ICP treatment modalities with corresponding scores for intensity, assessed daily [3] (Additional file 1: Supplement 1). High intensity ICP-lowering treatment is indicated by the use of one or more of the four treatments representing maximum therapy intensity on the TIL scale: Barbiturates (or high dose sedation) for metabolic (e.g. burst) suppression, secondary decompressive craniectomy, intensive hyperventilation to PaCO_2_ < 4 kPa, and hypothermia < 35 °C. We refer to patients who received any of these treatments at any point in time during their ICU stay as the ‘high TIL’ group. In addition, we excluded patients with decompressive craniectomy on day 1 (i.e. primary decompressive craniectomy) as such patients are likely to have a different pathophysiological trajectory and ICP therapy requirements due to a fundamental difference in intracranial compliance at the start of their ICU course. Such patients are also likely to be a distinct clinical entity (decompression at the time of space occupying lesion evacuation rather than for intractable intracranial hypertension) so their exclusion ensured a homogeneous population for a propensity score analysis. Maximum ICP prior to high TIL treatment (derived from 2 hourly measurements) was used as a measure of ICP burden.

### Outcomes

Outcomes were collected at 6-months post-injury. Functional outcome was assessed on the Glasgow Outcome Scale-Extended (GOSE) using either an interview or questionnaire. Categories on the GOSE are: (1) Death, (2) Vegetative State, (3) Lower Severe Disability, (4) Upper Severe Disability, (5) Lower Moderate Disability, (6) Upper Moderate Disability, (7) Lower Good Recovery, and (8) Upper Good Recovery. Patients in categories (2) and (3) on the GOSE were combined in a single category. Health related quality of life (HRQOL) was assessed with the Short Form 36v2 (SF-36) and the Quality of Life after Brain Injury (QOLIBRI) scale. For the SF-36, the Physical Component Summary (PCS) and Mental Component Summary (MCS) are expressed as T-scores. The QOLIBRI Total score has a range from 0 to 100.

### Statistical analyses

We stratified the high- and low TIL treatment group and described their baseline characteristics and outcome by frequency/percentages for categorical variables and by median and interquartile ranges (IQR) for continuous variables. Significant group differences were determined with the χ^2^ or Fisher’s exact test for categorical variables, and ANOVA or Kruskal Wallis test (non-normal distributions) for continuous variables.

Missing data were imputed using multiple imputation (100 imputations, 5 iterations) using the MICE package for R statistical software (version 3.6.0) [11]. The distribution of missingness per variable (prior to imputation) is shown in Additional file 2: Supplement 2.

To calculate the between-centre variation in the use of high TIL therapies beyond that expected from case-mix severity and random variation, we used a random effects logistic regression model, with high TIL use as dependent variable and centre as random intercept. Covariates used were chosen from the extended International Mission for Prognosis and Analysis of Clinical Trials (IMPACT) prognostic model [12]. In addition, we adjusted for maximum recorded ICP values prior to high TIL treatment (as a surrogate for prior secondary injury and/or difficulty in achieving control), CT variables likely to be associated with the development of intracranial hypertension (brain herniation, cortical sulcus effacement, compression of one of more basal cisterns, midline shift and ventricular compression), as well as extracranial injury severity score (ISS; excluding the head injury component). Centre effects are expressed and plotted as random effects with corresponding confidence intervals at a log odds scale. We also quantified the between-centre variation with the median odds ratio (MOR): The MOR is a measure of the variance of the random effects [13]. The Nakagawa's *R*^2^ for mixed models was calculated to determine the variance in high TIL treatment explained by the variables in the model.

In previous studies, the aggressiveness of TBI management has been quantified based on the percentage use of ICP monitoring in patients who satisfied Brain Trauma Foundation (BTF) guidelines requirements for such monitoring. In order to examine whether this definition of aggressiveness based on use of a monitoring modality actually translated into aggressive management, we examined whether the percentage use of ICP monitoring in centres was related to the random effects of the use of high TIL per in the centre.

Finally, to study the association between high TIL treatment use and outcome, a propensity score matched model was constructed. This analysis determines whether the application of any high TIL therapy resulted in incremental harm (aggressiveness of treatment) beyond that caused by ICP elevation and case-mix severity. The primary outcome was the Glasgow Outcome Score-extended (GOSE) at 6 months, dichotomized into favourable (GOSE > 4) and unfavourable (GOSE ≤ 4). We used the random effects logistic regression model above to determine propensity scores for high TIL use. We applied nearest neighbour matching to select patients with a similar propensity scores but different treatment status. We compared the baseline characteristics between matched cases (with no missing data) and tested group differences (should be non-significant) and calculated the standardized mean differences (which should be low) to check match validity. In the matched cases, we compared the result of high versus low TIL treatment using a logistic regression with 6-month unfavourable GOSE as primary outcome. Two sensitivity analyses were performed to check whether the treatments were applied appropriately as high TIL practice. The first of these excluded high TIL treatments on day 1 to more faithfully reflect escalation of ICP therapy and discard non-treatment confounds (for example, hypothermia on day 1 may be injury related). Secondly, we considered the possibility that the use of barbiturates may have simply reflected therapy to target early / transient difficulties in controlling in ICP, rather than a sustained escalation of therapy. Consequently, the second sensitivity analysis excluded all barbiturate use as a high TIL treatment.

Analyses were performed using R statistical software [14]. The dataset was stored and accessed using the Opal [15] datamart. Dataset downloaded 06–02-2020 (Neurobot release 2.1).

## Results

### Baseline characteristics

**Fig. 1:**
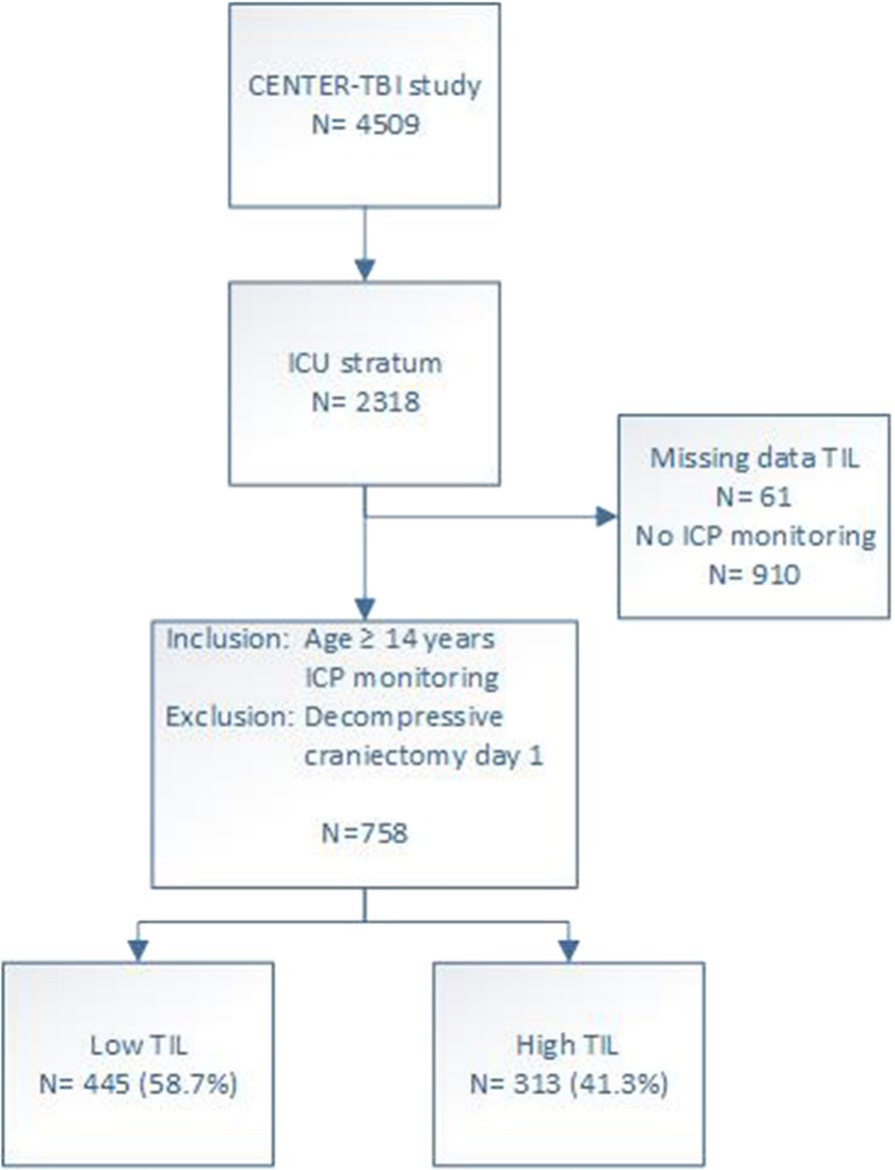
Flowchart: patient inclusion. This flowchart is showing the inclusion of high TIL patients. High TIL patients were defined as patients receiving any treatment during ICU stay representing maximum therapy intensity of the TIL scale: Barbiturates for metabolic suppression, (secondary) decompressive craniectomy, intensive hyperventilation to PaCO2 < 4 kPa, and hypothermia < 35 °C at any point during their ICU stay

A total of 758 patients from 52 centres in Europe received ICP monitoring with documented TIL measurements during their ICU stay (Fig. 1, Additional file 4: Supplement 4, Additional file 6: Supplement 6). Of these, 313 patients (41.3%) received high TIL treatments at least once during their ICU stay. Table 1 summarises these groups. Patients who received high TIL treatment were generally younger, had better preinjury health status, and suffered from more severe brain trauma. Multimodal cerebral monitoring was generally more often used in high TIL patients.

**Table 1:** Baseline patient and monitoring characteristics

	Low TIL(*N* = 445)	High TIL (*N* = 313)	*p*-value
Age (median, IQR)	51 [30.0–65.0]	41 [27.0–65.0]	< 0.001
Gender, male (*N*,%)	326/445 (73.3)	235/313 (75.1)	0.632
*Injury severity (GCS at baseline)*			
Mild > = 13 (*N*, %)	77/414 (18.6)	29/296 (9.8)	0.002
Moderate > = 9—< 13(*N*, %)	78/414 (18.8)	51/296 (17.2)	0.653
Severe < 9 (*N*, %)	259/414 (62.6)	216/296 (73.0)	0.005
ISS without head injury (median, IQR)	10 [0.0–25.0]	13 [0.0–25.0]	0.286
*CT (N,%)*			
tSAH	306/367 (− 83.4)	228/253 (− 90.1)	0.023
EDH	73/366 (− 19.9)	58/254 (− 22.8)	0.443
Marshall (*N*, %)			
1	11/367 (− 3)	5/253 (− 2)	< 0.001
2	168/367 (− 45.8)	94/253 (− 37.2)	
3	30/367 (− 8.2)	57/253 (− 22.5)	
4	8/367 (− 2.2)	5/253 (− 2)	
5	2/367 (− 0.5)	2/253 (− 0.8)	
6	148/367 (− 40.3)	90/253 (− 35.6)	
Preinjury ASA (*N*, %)			0.001
1) Normal healthy	230/428 (− 53.7)	194/296 (− 65.5)	
2) Mild systemic disease	144/428 (− 33.6)	88/296 (− 29.7)	
3) Severe systemic disease	51/428 (− 11.9)	13/296 (− 4.4)	
4) Severe systemic disease, a constant threat to life	3/428 (− 0.7)	1/296 (− 0.3)	
Cause of injury (*N*, %)			0.295
Road traffic incident	212/430 (− 49.3)	148/301 (− 49.2)	
Incidental fall	166/430 (− 38.6)	106/301 (− 35.2)	
Violence/assault	15/430 (− 3.5)	18/301 (− 6)	
Suicide attempt	10/430 (− 2.3)	4/301 (− 1.3)	
Other	27/430 (− 6.3)	25/301 (− 8.3)	
Prehospital^1^ (*N*, %)			
Hypoxia	89/445 (− 20)	39/313 (− 12.5)	0.009
Hypotension	80/445 (− 18)	47/313 (− 15)	0.329
Lab^1^ (median, IQR)			
Hemoglobin (g/dL)	13.1 [11.6–14.4]	13.2 [11.9–14.3]	0.694
Glucose (mmol/L)	7.9 [6.7–9.9]	7.8 [ 6.7–9.8]	0.933
Type ICP monitor (*N*, %)			0.511
Ventricular	53/445 (− 11.9)	38/312 (− 12.2)	
Ventricular + inbuilt sensor	7/445 (− 1.6)	10/312 (− 3.2)	
Parenchymal	366/445 (− 82.2)	250/312 (− 80.1)	
Other	19/445 (− 4.3)	14/312 (− 4.5)	
Multimodal cerebral monitoring (*N*, %)			
Jugular oximetry	9/445 (− 2)	20/312(− 6.4)	0.004
Brain tissue *P*_bt_O_2_	84/445 (− 18.9)	65/312 (− 20.8)	0.555
Transcranial Doppler	34/444 (− 7.7)	78/312 (− 25)	< 0.001
Microdialysis	48/445 (− 10.8)	30/311 (− 9.6)	0.7
Continuous EEG	21/445 (− 4.7)	38/311 (− 12.2)	< 0.001
Electrocorticography	4/444 (− 0.9)	1/311 (− 0.3)	0.61
Systemic monitoring (N, %)			
Invasive blood pressure monitoring	432/445 (− 97.1)	306/312 (− 98.1)	0.53
Cardiac output	74/444 (− 16.7)	80/312 (− 25.6)	0.003
Pulse oximetry	436/445 (− 98)	303/312 (− 97.1)	0.6
End tidal CO_2_	335/444 (− 75.5)	213/312 (− 68.3)	0.036
Central venous pressure	261/444 (− 58.8)	190/312 (− 60.9)	0.612
Mechanical ventilation			
Present	416/441 (− 94.3)	287/312 (− 92)	0.261

This table describes the baseline characteristics of patients with a high versus a low therapy intensity level (TIL). High TIL was defined as any high intensity treatment (decompressive craniectomy excluding day 1, barbiturates, intensive hypothermia, intensive hyperventilation) during the ICU stay. Significant group differences were determined by using the chi-square or Fisher’s exact test (non-normal distributions) for categorical variables and an ANOVAS or Kruskal Wallis test (non-normal distributions) for continuous variables

^1^IMPACT, first available

ASA score: American Society of Anesthesiologists (ASA) Physical Status score, CO_2_: carbon dioxide, CT: Computed tomography, CRBSI: catheter-related blood-stream infection EEG: electroencephalogram, GCS: Glasgow Coma Scale, ICP: intracranial pressure, ISS: injury severity score, IQR: interquartile range, *P*_bt_O_2_: brain tissue partial pressure of oxygen, TIL: therapy intensity level

Patients requiring high TIL treatment generally had longer ICU stays and had a longer duration of mechanical ventilation (Table 2). Overall, high TIL and low TIL patients were discharged from the ICU with similar GCS scores. The complication rate was similar in the two groups, except for metabolic complications (high TIL: 14.0%, versus low TIL: 7.3% *p* = 0.004) (abnormalities in renal or liver function and electrolyte derangements).

**Table 2:** Patient outcomes

	Low TIL (*N* = 445)	High TIL (*N* = 313)	*p*-value
General ICU outcomes (median, IQR)			
Length of ICU stay	11 [5.8–19]	17 [10–26]	< 0.001
Duration of ventilation	8 [4.0–15]	14 [8–21]	< 0.001
Time to obey commands	6 [2.0–11]	13 [21]	< 0.001
ICU systemic complications (*N*, %)			
Cardiovascular	52/441 (− 11.8)	34/307 (− 11.1)	0.853
CRBSI	20/441 (− 4.5)	7/307 (− 2.3)	0.154
DVT	5/441 (− 1.1)	7/307 (− 2.3)	0.351
Pulmonary embolus	10/441 (− 2.3)	5/307 (− 1.6)	0.728
Metabolic^1^	32/441 (− 7.3)	43/307 (− 14)	0.004
Pressure sores	20/441(− 4.5)	15/307 (− 4.9)	0.962
Respiratory failure	156/441 (− 35.4)	123/307 (− 40.1)	0.219
VAP	107/441 (− 24.3)	85/307 (− 27.7)	0.332
UTI	36/441 (− 8.2)	30/307 (− 9.8)	0.527
Other	38/441 (− 8.6)	32/307 (− 10.4)	0.48
ICU discharge outcomes			
ICU mortality (*N*, %)	59/442 (− 13.3)	62/307 (− 20.2)	0.016
GCS discharge score			
Mild > = 13 (*N*, %)	121/445 (− 27.2)	75/313 (− 24)	0.36
Moderate > = 9—< 13(*N*, %)	13/445 (− 2.9)	13/313 (− 4.2)	0.475
Severe < 9 (*N*, %)	311/445 (− 69.9)	225/313 (− 71.9)	0.607
Outcomes after 6 months			
GOSE	344 /381 (− 90.3)	262/280 (− 93.6)	0.171
GOSE < 8 (*N*, %)	202/381 (− 53)	175/280 (− 62.5)	0.019
GOSE < 5 (*N*, %)	107/381 (− 28.1)	63/280 (− 22.6)	
GOSE = 1 (N, %)			
Qolibri			
Impaired (< 52) (*N*, %)	32/ 173 (− 18.5)	29/121 (− 24)	0.321
SF-36 MCS			
Impaired (< 40) (*N*, %)	57/173 (− 32.9)	40/117 (− 34.2)	0.926
SF-36 PCS			
Impaired (< 40) (*N*, %)	57/173 (− 32.9)	40/117 (− 34.2)	0.926

This table describes the outcomes of patients stratified by high versus a low therapy intensity level treatment. High TIL was defined as any high intensity treatment (decompressive craniectomy excluding day 1, barbiturates, intensive hypothermia, intensive hyperventilation) during ICU stay. Significant group differences were determined by using the chi-square or Fisher’s exact test (non-normal distributions) for categorical variables and an ANOVA or Kruskal Wallis test (non-normal distributions) for continuous variables

^1^Significant variation in proportion of abnormal lab values for high TIL vs. low TIL patients, i.e. creatinine (24.8% vs. 20.1%, *p* < 0.001), sodium (50.1% vs. 39.7%, *p* < 0.001), ASAT (49.1% vs 49.2%, *p* = 0.865), and ALAT (39.3% vs. 37.9%, *p* = 0.020)

CRBSI: Catheter-related bloodstream infection, DVT: Deep Venous Thrombosis, GCS: Glasgow Coma Scale, GOSE: Glasgow Outcome Scale Extended, ICU: Intensive Care Unit, Qolibri: Quality of life after brain injury total score, SF-36 MCS: Short Form-36v2 Mental Component Summary, SF-36 PCS: Short Form-36v2 Physical Component Summary, TIL: therapy intensity level, VAP: ventilator acquired pneumonia

### Patterns of high TIL therapy use

Of the 313 patients, most received metabolic suppression while a minority of cases received intensive hyperventilation, intensive hypothermia, or secondary decompressive craniectomy (Additional file 3: Supplement 3, Additional file 5: Supplement 5). In general, TIL peaked after day 2, except for hypothermia (which was most commonly applied on day 1). In the majority of cases receiving high TIL treatment, head elevation, vasopressors and higher dose sedation had been used, but cerebrospinal fluid (CSF) drainage, hyperosmolar therapies, and being nursed flat were recorded only in a minority of instances. Mean TIL scores in the high TIL group were below 10 points.

### Between centre variation

**Fig. 2:**
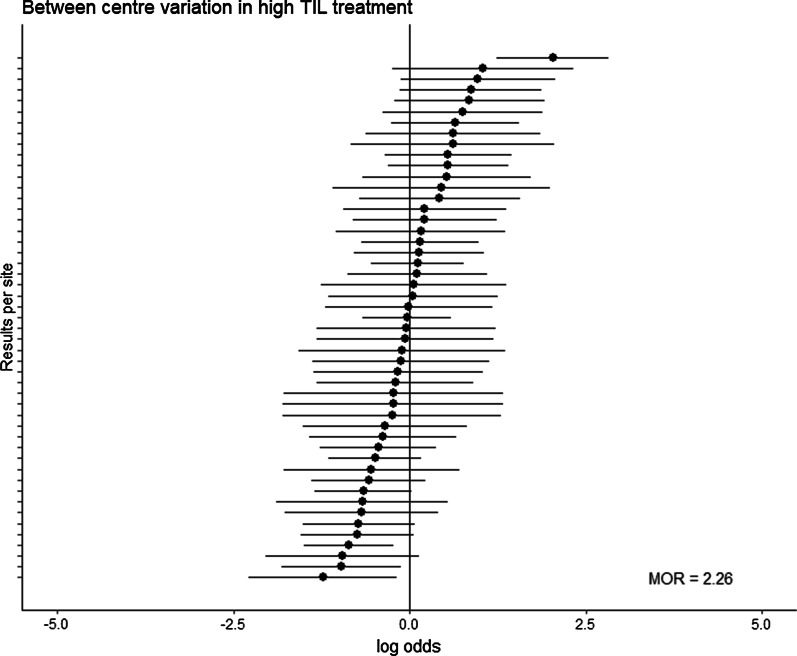
Between-centre variation in high TIL use. This figure shows the between-centre variation in the use of high TIL (Therapy Intensity Level) treatment. The use of high TIL per centre was adjusted for case-mix severity, brain herniation on imaging, maximum ICP value at the day of the start of high TIL treatment and random variation per centre with a random effects logistic regression model. For each centre, the random effect with corresponding 95% CI is plotted (average effect is log odds of zero). The MOR reflects the odds of high TIL treatment between two randomly selected centres for patients with the same case-mix severity (a higher MOR reflects larger between-centre variation) The MOR represents the median odds ratio; the higher the MOR the larger the between-centre variation (a MOR of 1 reflects no variation)

**Fig. 3:**
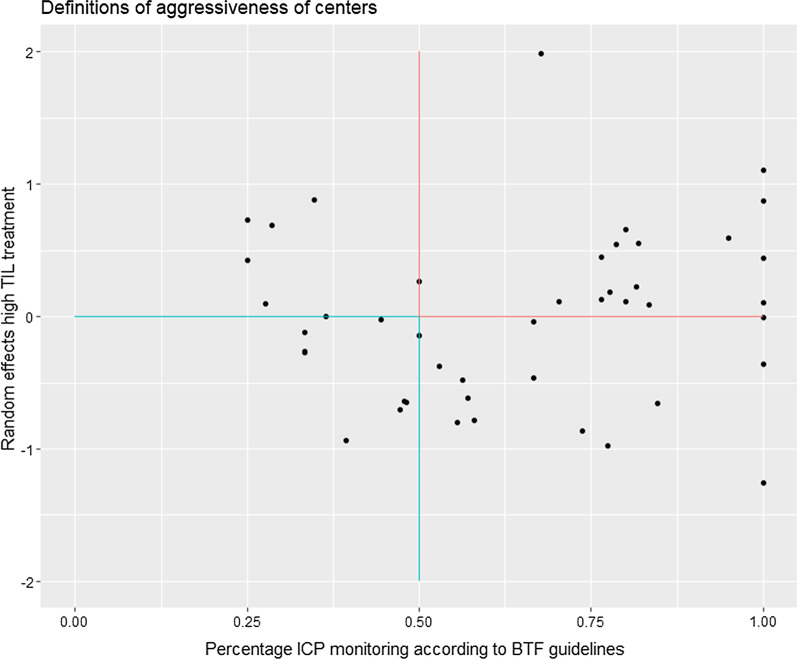
Definitions of aggressiveness. This figure illustrates the concordance between two definitions to identify aggressiveness of centers. On the x-axis is the definition of aggressiveness according to previous studies: the percentage of patients receiving ICP monitoring according to the BTF guidelines (GCS < 8 and abnormal CT, or normal CT and 2 or more of the following: hypotension, age > 40 years, unilateral or bilateral motor posturing, or systolic blood pressure (BP) < 90 mmHg). This percentage ICP monitoring was calculated in the ICU database (including all patients). On the y-axis is the definition of aggressiveness according to our study: the random effects of high TIL treatment per centers (log odds of receiving high TIL treatment). The upper right quadrant shows the centers that are both identified as aggressive by the previous definition (threshold 50% ICP monitoring) and the definition in our study (threshold random effect of zero).The lower left quadrant shows the centers that are identified as non-aggressive centers by both definitions. The two other quadrants show a discrepancy between the definitions of aggressiveness. Overall, there is no relationship between aggressiveness defined using ICP monitoring rates and actual use of aggressive therapies for ICP control

Our study included 52 centres from 18 countries in the CENTER-TBI study. The median number of patients per centre was 11.5 [IQR 5–19]. Most centres used barbiturates (*N* = 46) while fewer centres used intensive hyperventilation (*N* = 21), hypothermia below 35 °C (*N* = 32), and decompressive craniectomy (*N* = 26). Based on treatment frequencies, there was a high degree of between centre variation in treatment choice. Overall, significant between centre variation beyond case mix and random variation (*p* < 0.001) was found in the use of high TIL treatments (MOR = 2.26). (Fig. 2, Additional file 7: Supplement 7). The Nakagawa's *R*^2^ showed that model variables ‘explained’ 8.7% of the (pseudo)variance in high TIL treatment use. Comparing measures of aggressiveness, the percentage use of ICP monitoring in patients who satisfied BTF guidelines was not related to the use of high TIL therapies by the centre (Fig. 3).

### Impact of high TIL treatment on outcome

**Fig. 4:**
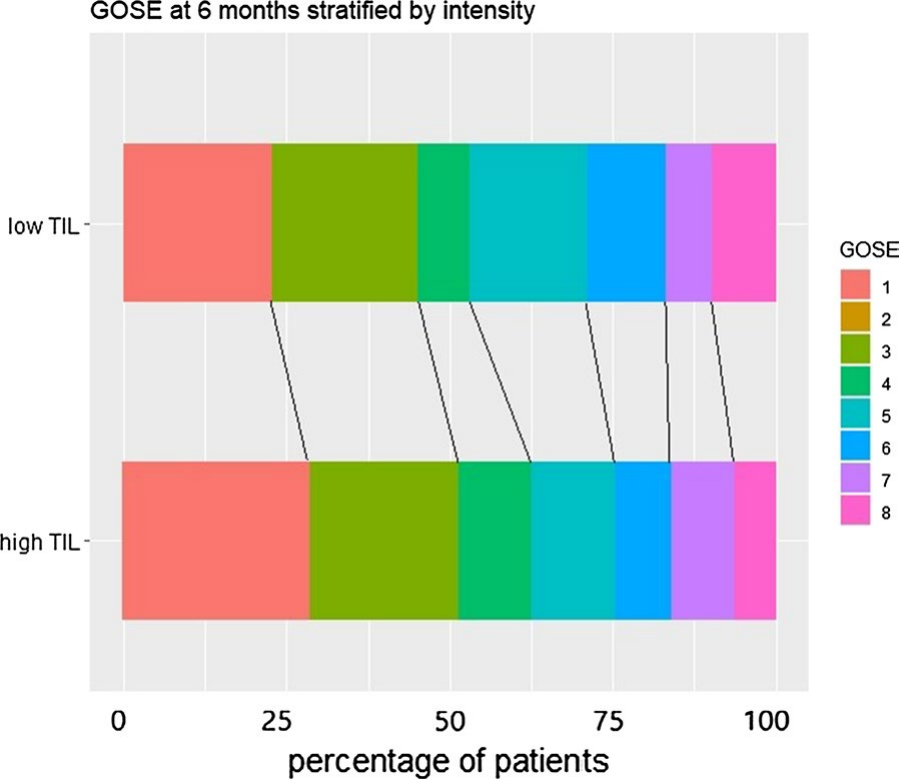
Functional outcome at 6 months. This figure shows the functional outcome (GOSE) at 6 months for patients who receive low therapy and high therapy intensity. GOSE 1: death, 2: vegetative state, 3: severe disability lower, 4: severe disability upper, 5: moderate disability lower, 6: moderate disability upper, 7: good recovery lower, 8: good recovery upper. Patients in categories (2) and (3) on the GOSE were combined in a single category. GOSE: Glasgow Outcome Scale Extended, TIL: Therapy Intensity Level were combined in a single category. GOSE: Glasgow Outcome Scale Extended, TIL: Therapy Intensity Level

Although unfavourable outcome was more frequent in the high TIL group (62.5% versus 53.0%, *p* = 0.019)–a high proportion of high TIL patients nevertheless achieved a favourable outcome at 6 months (GOSE ≥ 5: *N* = 105; 37.5%). Mortality was significantly higher in the high TIL group (20.2% versus 13.3%, *p* = 0.016) (Fig. 4). The data on Health-Related Quality of Life (HRQOL) are less complete than the GOSE, since in addition to loss to follow-up there are no scores for patients who die. Both groups had similar scores on the SF-36v2 MCS and PCS and the QOLIBRI total score. (Table 2).

A total of 280 treated (high TIL) patients were well matched in terms of their baseline characteristics (Additional file 8: Supplement 8) and maximum ICP prior to TIL treatment did not differ between groups (Additional file 9: Supplement 9). With correction for maximum ICP prior to high TIL treatment; high TIL treatment was not significantly associated with unfavourable outcome (OR 1.4, 95% CI [0.98–1.96], *p* = 0.068). However, after the sensitivity analyses the association with worse outcome became significant for high TIL after day 1 (OR 1.5 CI [1.1–2.2], *p* = 0.023) and high TIL excluding barbiturates (OR 2.5 CI [1.4–4.7] *p* = 0.004). (Table 3).

**Table 3:** Adjusted outcome after high TIL versus low TIL treatment (propensity score matched model)

Main analysis				Sensitivity analyses					
High TIL treatment				High TIL treatment after day 1			High TIL treatment excluding barbiturates		
	Nr	OR [95% CI]	*p*	Nr	OR [95% CI]	*p*	Nr	OR [95% CI]	*p*
Unfavourable GOSE ≤ 4	280	1.4 [1.0–2.0]	0.068	250	1.5 [1.1–2.2]	0.023	114	2.5 [1.4–4.7]	0.004

This table describes the differences in outcome after receiving high Therapy Intensity Level (TIL) treatment versus low TIL treatment. Overall high TIL was defined as any high intensity treatment (decompressive craniectomy excluding day 1, barbiturates, intensive hypothermia, intensive hyperventilation) during ICU stay. The primary outcome is the GOSE after 6 months. We used a multivariate propensity score matched model with correction for centre effects (random intercept). Nearest neighbour matching was used to select patients with the similar propensity scores and different treatment status. Covariates used for matching were IMPACT variables and ‘ISS without head injury ‘and in the final model we corrected for maximum ICP values prior to treatment. For the sensitivity analyses, we only selected patient with high TIL treatments after day 1 or excluded patients receiving barbiturates

CI: confidence interval, GOSE: Glasgow Outcome Scale Extended, ICP: intracranial pressure, ICU: intensive care unit, Nr: number of matches, OR: odds ratio, TIL: therapy intensity level

## Discussion

To our knowledge, this is the first study to quantify treatments using the TIL scale in real-world clinical practice across centres in Europe. We report substantial between-centre variation in the choice and use of high TIL treatments in patients with TBI admitted to the ICU across Europe. Further, we did not observe a systematic progression in therapy intensity, exhausting low-tier treatments before escalating to more intensive therapies: instead high tier therapies were often used early in the course of treatment. This was unexpected, because progressive approach to treatment is recommended by the Brain Trauma Foundation guidelines [16] and forms part of the standard protocol in previous large clinical trials. In line with previous observational studies, we found relatively infrequent use of intensive hyperventilation, or decompressive craniectomy [17, 18]. In contrast, we found a relatively liberal use of barbiturates/deep sedation for metabolic suppression [19]. We found significant between centre variation in high TIL therapy use, beyond that accounted for by case-mix severity and random variation, both in terms of choice of therapy (e.g. use of hypothermia in a centre) and overall frequency of use (corrected for case-mix severity and random variation). This variation in high TIL treatment at centre-level suggests that, apart from disease severity, the clinical decision to use high TIL treatment is also based on institutional policy and culture.

After correction for ICP control, no statistically significant association was found between the use of high TIL treatment and functional outcome at 6 months. However, when excluding high TIL treatment at day 1 or barbiturates from high TIL treatment there was a statistically significant association with worse outcome. This may reflect some unquantified aspect of disease severity that is not captured by the available covariates but nevertheless translates into both TIL and outcome differences. Alternatively, this could mean that there is indeed some harm from residual high TIL therapies, in which case the use of these therapies before less hazardous low TIL options are exhausted could expose patients to unnecessary risks. Still, high-level evidence is lacking about the use of individual lower TIL therapies like CSF drainage and hyperosmolar fluids. This might explain why centres are cautious to apply these lower TIL treatments as standard use before proceeding to higher TIL treatment. Future studies are needed to confirm these findings as the sample size might have been insufficient to detect an association and to determine if a certain patient subgroup might benefit. High TIL treatments were associated with increased duration of ventilation and longer lengths of stay although we did not find a higher complication rate, at least for the metrics recorded. While we matched the two groups on available factors known to influence outcome, it is also possible that other aspects of the clinical course which we could not capture are also important in a clinician’s decision to institute high TIL therapies (residual confounding).

An important finding is that a large proportion of patients receiving high TIL treatments nevertheless recovered to good functional outcome (moderate disability to good recovery) at 6 months. High TIL treatment might be an appropriate final resource for patients with refractory high ICP values and may be beneficial in this group. Nevertheless, since there could be risks of such treatments, this emphasises the need for their rational use. More work is required to understand if outcome benefits could result from a more consistently gradated and progressive application of treatment intensity and/or a shift from institutional policies towards individualized medicine.

Previous studies have defined highly intensive (aggressive) treatment for ICP control in different ways [4–7, 20]. Cnossen et al*.* explored various definitions for aggressive treatment, such as the definitions ‘use of ICP monitoring in more than 50% in patients meeting the BTF guidelines criteria’ and ‘aggressiveness based on a TIL score (any of the following: osmotic therapy, hyperventilation, cerebrospinal fluid drainage, vasopressors for cerebral perfusion pressure support, hypothermia, barbiturates, and neurosurgical intervention)’ [4]. Bulger et al*.* also defined aggressiveness as ‘the use of ICP monitoring according to the BTF guidelines in more than 50% of patients’ [5]. However, this definition of aggressiveness (use of ICP monitoring) did not correlate with measured aggressiveness of therapy in our study, defined in our dataset as the likelihood of using high TIL therapies. We conclude that the previous use of higher use of ICP monitoring as a marker of aggressive TBI management in a centre may be flawed.

Several recent large trials have studied the impact on outcome of individual high TIL treatments, such as decompressive craniectomy [6, 8] or intensive hypothermia [21], but there is a need to assess other hazardous ICP-directed therapies (such as intensive hyperventilation and barbiturate coma) in this setting [16]. Our analysis targeted integrated assessment of all of these therapies, but the heterogeneity and lack of a uniform tiered approach to their use suggest that comparative effectiveness research (CER) approaches to exploring these therapies may have problems.

This study has a number of limitations that need to be discussed. First, the definition of a high TIL treatment is to some extent arbitrary as it is based on expert opinion rather than concrete outcome data. We considered metabolic suppression as a second-tier treatment, based on the recommendation in BTF guidelines that barbiturates should be considered a second-tier therapy (for raised ICP refractory to maximum treatment) [16]. However, our data suggest that in many centres others might consider this a first-tier/early therapy, in keeping with results from our Provider Profiling exercise [22]. In addition, we have no data on whether short durations of metabolic suppression in the early phase of illness carry the same risks as prolonged metabolic suppression employed as a treatment for refractory ICP in a later stage. Second, we do not have detailed data on how carefully these treatments were implemented, which is a significant omission. For example, the methods and rates of cooling or re-warming could affect both the efficacy and harm associated with intensive hypothermia. Finally, incomplete data on ICP monitoring made it difficult to accurately define a metric for poor ICP control before escalation of therapy and hence made propensity matching difficult. As poorly controlled ICP is likely to be a driver for escalation of therapy (or for continuing high TIL therapy), and also a marker of poor outcome, the absence of these data makes a rigorous covariate-adjusted comparison of high and low- TIL therapy groups difficult.

### Future directions

Further work will be needed to explore the process by which clinical decisions to proceed to more intensive treatments are undertaken and determine the best way that hazardous therapies should be introduced in a rational tiered treatment plan. The evidence base to choose a particular high TIL treatment over another is limited, since the evidence on benefit from these therapies is either absent or conflicting [6–8, 21]. This lack of evidence helps to explain high between-centre variation in choice of treatment, and currently means that the initiation and choice of high TIL interventions is only driven by patient characteristics to a very limited extent and is primarily based on institutional policies. A better identification of subgroups of patients who benefit from such therapies would allow better targeting of either individual interventions, or high intensity therapies in general. We also need to explore whether more rigorous ICP control, with higher intensity therapies, may, in a subgroup of patients, prevent refractory intracranial hypertension, reduce ICU stay, and possibly improve outcome. The search for patient and monitoring characteristics that identify such a subgroup could allow a precision medicine approach to ICP management.

## Conclusions

We show substantial variation amongst European centres in the choice and use of ICP-lowering treatments for patients with TBI. We found a no statistically significant association between the use of high TIL therapies and worse outcome after 6 months although a significant association did become apparent when day 1 or high dose sedation was excluded. However, this difference may have been flawed because of incomplete propensity matching of the high TIL and control groups due to unmeasured covariates. In any case, our results do not support a nihilistic view of patients who receive high TIL treatments; one third of high TIL patients achieved a favourable functional outcome, and high TIL treatment might have contributed to this. Further studies need to confirm whether and when high TIL treatments can be used as a safe final resort. More consistent use of low-tier treatments before escalating management to high TIL therapies, and data that support a rational choice of high TIL therapies, could both contribute to improved clinical outcome.

## Supplementary information

**Additional file 1.** Therapy Intensity Level scale. Description: This table shows the scoring of the Therapy Intensity Level (TIL) as recorded in the CENTER-TBI study. Derived from Zuercher et al. [3]. High TIL therapies are shown in bold.

**Additional file 2.** Missing data. Description: This figure shows the proportion of missing data in the original data (before imputation). In the left panel the proportion of missingness per variable is shown. In the combination plot (grid) all patterns of missing (red) and observed data (blue) are shown. For example, the bottom row shows all patients with complete data, above that the patients with the combination missing data for Hb and gluc, ect. The bars on the right of the combination plot show the frequency of occurrence of the combinations.

**Additional file 3.** Treatments on the TIL scale (all patients).Description: This table shows the number and percentages of ICP-monitored patients receiving ICP-lowering treatments on the TIL scale. Each row shows the number of patients (frequency) that receive that treatment (a patient could receive multiple treatments per topic).

**Additional file 4.** Daily TIL scores. Description: This figure shows the daily high TIL scores (cumulative score of the high TIL treatments) plotted against the daily low TIL scores (cumulative score for low TIL treatments). It shows that at the same high TIL scores a variety of low TIL treatment (scores) is applied (in some cases even no low TIL treatment). Also, the figure shows mainly at day 3 (dark green) higher TIL treatments are applied including higher low TIL scores.

**Additional file 5.** Treatments on the TIL scale (high TIL patients). Description: This table shows the number and percentages of high TIL patients receiving ICP-lowering treatments on the TIL scale. Each row shows the number of patients (frequency) that receive that treatment (a patient could receive multiple treatments per topic) Bolt treatment are regarded as high TIL treatment.

**Additional file 6.** Higher tier ICP-lowering treatments in patients receiving high TIL treatment. Description: This figure shows the proportion of patients that receive first and second tier treatments of the high TIL patients across 7 days at the Intensive Care Unit.

**Additional file 7.** Variation in high TIL treatment percentages across centres (centre-level). Description: This table describes the between-centre variation for high TIL treatments across the days. The number of centres are the centres that actually apply the individual treatments. The mean percentage represents the mean percentage of patients across centres that receive the therapy, while the IQR and min-max represents the variation in treatment use between centres.

**Additional file 8.** Baseline characteristics matched dataset. Description: This table describes the baseline characteristics of the matched cases with complete data (as the dataset was imputed, this table could only be completed for complete cases). Significant group differences were determined by using the chi-square or Fisher’s exact test (non-normal distributions) for categorical variables and an ANOVA or Kruskal Wallis test (non-normal distributions) for continuous variables.

**Additional file 9.** Maximum ICP values prior to start high TIL use per treatment group. Description: This figure shows the differences in maximum ICP values prior to high TIL treatment between patients with a high TIL (1) versus a low TIL treatment (0). The median ICP for low TIL is 22 [16-28] and for high TIL 22 [16-27]. This difference is not statistically significant.

## Data Availability

The datasets used and/or analyzed during the current study are available via https://www.center-tbi.eu/data on reasonable request.

## Abbreviations

CENTER-TBI: Collaborative European NeuroTrauma Effectiveness Research in Traumatic Brain Injury
CER: Comparative effectiveness research
CSF: Cerebrospinal fluid;
CT: Computed tomography
GCS: Glasgow Coma Scale
GOSE: Glasgow Outcome Scale Extended
HRQOL: Health-Related Quality of Life
ICP: Intracranial pressure
ICU: Intensive care unit
IMPACT: International Mission for Prognosis and Analysis of Clinical Trials
ISS: Injury Severity Score
MOR: Median odds ratio
OR: Odds ratio
TIL: Therapy Intensity Level
TBI: Traumatic brain injury

## References

[1] Maset AL, Marmarou A, Ward JD, Choi S, Lutz HA, Brooks D, Moulton RJ, DeSalles A, Muizelaar JP, Turner H (1987). Pressure-volume index in head injury. J Neurosurg.

[2] Shore PM, Hand LL, Roy L, Trivedi P, Kochanek PM, Adelson PD (2006). Reliability and validity of the pediatric intensity level of therapy (PILOT) scale: a measure of the use of intracranial pressure-directed therapies. Crit Care Med.

[3] Zuercher P, Groen JL, Aries MJ, Steyerberg EW, Maas AI, Ercole A, Menon DK (2016). Reliability and validity of the therapy intensity level scale: analysis of clinimetric properties of a novel approach to assess management of intracranial pressure in traumatic brain injury. J Neurotrauma.

[4] Cnossen MC, Polinder S, Andriessen TM, van der Naalt J, Haitsma I, Horn J, Franschman G, Vos PE, Steyerberg EW, Lingsma H (2017). Causes and consequences of treatment variation in moderate and severe traumatic brain injury: a multicenter study. Crit Care Med.

[5] Bulger EM, Nathens AB, Rivara FP, Moore M, MacKenzie EJ, Jurkovich GJ, Brain Trauma F (2002). Management of severe head injury: institutional variations in care and effect on outcome. Crit Care Med.

[6] Hutchinson PJ, Kolias AG, Timofeev IS, Corteen EA, Czosnyka M, Timothy J, Anderson I, Bulters DO, Belli A, Eynon CA (2016). Trial of decompressive craniectomy for traumatic intracranial hypertension. N Engl J Med.

[7] Cooper DJ, Nichol AD, Bailey M, Bernard S, Cameron PA, Pili-Floury S, Forbes A, Gantner D, Higgins AM, Huet O (2018). Effect of early sustained prophylactic hypothermia on neurologic outcomes among patients with severe traumatic brain injury: the POLAR randomized clinical trial. JAMA.

[8] Cooper DJ, Rosenfeld JV, Murray L, Arabi YM, Davies AR, D'Urso P, Kossmann T, Ponsford J, Seppelt I, Reilly P (2011). Decompressive craniectomy in diffuse traumatic brain injury. N Engl J Med.

[9] Steyerberg EW ea: The contemporary landscape of traumatic brain injury in Europe: Case-mix, care pathways, and outcomes from the CENTER-TBI study. *Lancet neurology* 2019. 10.1016/S1474-4422(19)30232-7 31526754

[10] Maas AI, Menon DK, Steyerberg EW, Citerio G, Lecky F, Manley GT, Hill S, Legrand V, Sorgner A, Participants C-T (2015). Collaborative European neurotrauma effectiveness research in traumatic brain injury (CENTER-TBI): a prospective longitudinal observational study. Neurosurgery.

[11] Van Buuren S. G-OK: mice: Multivariate Imputation by Chained Equations in R. J Stat Softw 45. 2011.

[12] Steyerberg EW, Mushkudiani N, Perel P, Butcher I, Lu J, McHugh GS, Murray GD, Marmarou A, Roberts I, Habbema JD (2008). Predicting outcome after traumatic brain injury: development and international validation of prognostic scores based on admission characteristics. PLoS Med.

[13] Merlo J, Chaix B, Ohlsson H, Beckman A, Johnell K, Hjerpe P, Rastam L, Larsen K (2006). A brief conceptual tutorial of multilevel analysis in social epidemiology: using measures of clustering in multilevel logistic regression to investigate contextual phenomena. J Epidemiol Community Health.

[14] Bonow RH, Quistberg A, Rivara FP, Vavilala MS (2019). Intensive care unit admission patterns for mild traumatic brain injury in the USA. Neurocrit Care.

[15] Doiron D, Marcon Y, Fortier I, Burton P, Ferretti V (2017). Software application profile: opal and mica: open-source software solutions for epidemiological data management, harmonization and dissemination. Int J Epidemiol.

[16] Brain Trauma F (2007). American association of neurological s, congress of neurological s: guidelines for the management of severe traumatic brain injury. J Neurotrauma.

[17] Neumann JO, Chambers IR, Citerio G, Enblad P, Gregson BA, Howells T, Mattern J, Nilsson P, Piper I, Ragauskas A (2008). The use of hyperventilation therapy after traumatic brain injury in Europe: an analysis of the BrainIT database. Intensive Care Med.

[18] Aarabi B, Hesdorffer DC, Ahn ES, Aresco C, Scalea TM, Eisenberg HM (2006). Outcome following decompressive craniectomy for malignant swelling due to severe head injury. J Neurosurg.

[19] Roberts I, Sydenham E. Barbiturates for acute traumatic brain injury. Cochrane Database Syst Rev 2012, **12**:CD000033. 10.1002/14651858.CD000033.pub2 PMC7061245 23235573

[20] Cooper DJ, Rosenfeld JV (2011). Does decompressive craniectomy improve outcomes in patients with diffuse traumatic brain injury?. Med J Aust.

[21] Andrews PJ, Sinclair HL, Rodriguez A, Harris BA, Battison CG, Rhodes JK, Murray GD, Eurotherm Trial C (2015). Hypothermia for intracranial hypertension after traumatic brain injury. N Engl J Med.

[22] Cnossen MC, Huijben JA, van der Jagt M, Volovici V, van Essen T, Polinder S, Nelson D, Ercole A, Stocchetti N, Citerio G (2017). Variation in monitoring and treatment policies for intracranial hypertension in traumatic brain injury: a survey in 66 neurotrauma centers participating in the CENTER-TBI study. Crit Care.

