# Supplementary material for: Use and impact of high intensity treatments in patients with traumatic brain injury across Europe: a CENTER-TBI analysis
Source: Crit Care. 2021 Feb 23;25:78. doi: 10.1186/s13054-020-03370-y (PMC7901510; doi:10.1186/s13054-020-03370-y)
Supplement: Supplementary file 1 — Additional file 1. Therapy Intensity Level scale. Description: This table shows the scoring of the Therapy Intensity Level (TIL) as recorded in the CENTER-TBI study. Derived from Zuercher et al. [3]. High TIL therapies are shown in bold. [file 13054_2020_3370_MOESM1_ESM.docx]

Additional file 1. Therapy Intensity Level scale

| Table 1. Therapy Intensity Level scale | | | |
| --- | --- | --- | --- |
| Item | Details | Score | Max |
| Positioning | - Head elevation for ICP control - Nursed flat (180°) for CPP management | 1  1 | 1 |
| Sedation and neuromuscular blockade | - Sedation (low dose as required for mechanical ventilation) - Higher dose sedation for ICP control (not aiming for burst suppression - **Metabolic suppression for ICP control with high dose barbiturates or propofol** - Neuromuscular blockade (paralysis) | 1  2  5  3 | 8 |
| CSF drainage | - CSF drainage low volume (<120 mL /day or < 5 mL/h) - CSF drainage high volume (≥ 120 mL/ day or ≥5 mL/h) | 2  3 | 3 |
| CPP management | - Fluid loading for maintenance of cerebral perfusion - Vasopressor therapy required for management of cerebral perfusion | 1  1 | 2 |
| Ventilatory management | - Mild hypocapnia for ICP control [PaCO2 4.6 - 5.3 kPa (35 - 40 mmHg)] - Moderate hyppocapnia for ICP control [PaCO2 4.0 - 4.5 kPa (30 - 35 mmHg)] - **Intensive hypocapnia for ICP control [PaCO2 < 4.0 kPa (<30 mmHg)]** | 1  2  4 | 4 |
| Hyperosmolar therapy | - Mannitol up to2 g/kg/24h - Mannitol (>2 g/kg/24h) - Hypertonic saline up to 0.3 g/kg/24h - Hypertonic saline (>0.3 g/kg/24h) | 2  3  2  3 | 6 |
| Temperature control | - Treatment of fever (temperature > 38°C or spontaneous temperature of 34.5°C) - Cooling for ICP control with a lower limit of 35°C - **Hypothermia below 35°C** | 1  2  5 | 5 |
| Surgery for intracranial hypertension | - Intracranial operation for progressive mass lesion, not scheduled on admission - **Decompressive craniectiomy** | 4  5 | 9 |
| Maximum possible score | | | 38 |
| This table shows the scoring of the Therapy Intensity Level (TIL) as recorded in the CENTER-TBI study. Derived from Zuercher et al. [3]. High TIL therapies are shown in bold | | | |
