# Supplementary material for: Use and impact of high intensity treatments in patients with traumatic brain injury across Europe: a CENTER-TBI analysis
Source: Crit Care. 2021 Feb 23;25:78. doi: 10.1186/s13054-020-03370-y (PMC7901510; doi:10.1186/s13054-020-03370-y)
Supplement: Supplementary file 2 — Additional file 2. Missing data. Description: This figure shows the proportion of missing data in the original data (before imputation). In the left panel the proportion of missingness per variable is shown. In the combination plot (grid) all patterns of missing (red) and observed data (blue) are shown. For example, the bottom row shows all patients with complete data, above that the patients with the combination missing data for Hb and gluc, ect. The bars on the right of the combination plot show the frequency of occurrence of the combinations. [file 13054_2020_3370_MOESM2_ESM.docx]

Additional file 2. Missing data


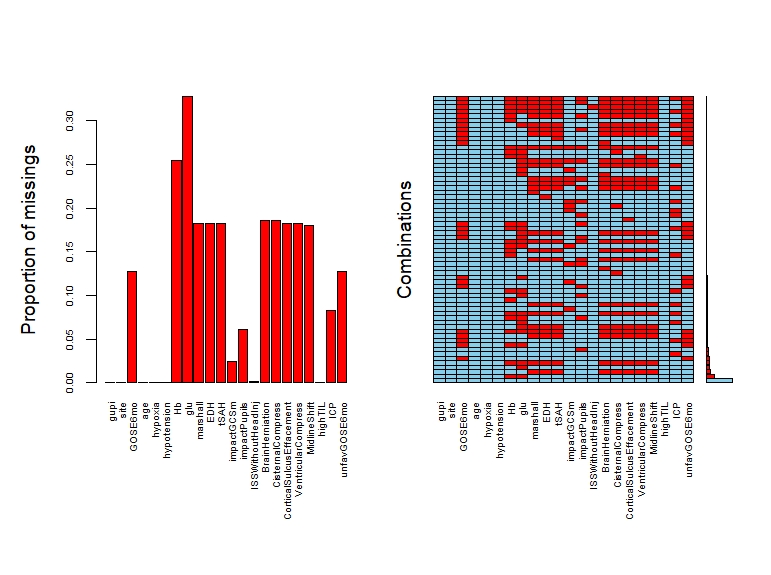


This figure shows the proportion of missing data in the original data (before imputation). In the left panel the proportion of missingness per variable is shown. In the combination plot (grid) all patterns of missing (red) and observed data (blue) are shown. For example, the bottom row shows all patients with complete data, above that the patients with the combination missing data for Hb and gluc, ect. The bars on the right of the combination plot show the frequency of occurrence of the combinations
