# Supplementary material for: Use and impact of high intensity treatments in patients with traumatic brain injury across Europe: a CENTER-TBI analysis
Source: Crit Care. 2021 Feb 23;25:78. doi: 10.1186/s13054-020-03370-y (PMC7901510; doi:10.1186/s13054-020-03370-y)
Supplement: Supplementary file 3 — Additional file 3. Treatments on the TIL scale (all patients).Description: This table shows the number and percentages of ICP-monitored patients receiving ICP-lowering treatments on the TIL scale. Each row shows the number of patients (frequency) that receive that treatment (a patient could receive multiple treatments per topic). [file 13054_2020_3370_MOESM3_ESM.docx]

Supplement 3. Treatments on the TIL scale (all patients)

| Table 2. Frequencies of treatments on the TIL scale (all patients) | | | | | | | | | | |
| --- | --- | --- | --- | --- | --- | --- | --- | --- | --- | --- |
| Days at the ICU  Total number patients | Day 1  745 | Day 2  742 | | Day 3  719 | Day 4  698 | | | Day 5  665 | Day 6  634 | Day 7  614 |
| Positioning |  |  | |  |  | | |  |  |  |
| - Head elevation | 608 (85.5) | 623 (87.0) | | 583 (84.1) | 551 (83.0) | | | 515 (81.6) | 488 (81.7) | 451 (78.6) |
| - Nursed flat | 75 (10.6) | 71 (9.9) | | 68 (9.8) | 62 (9.4) | | | 55 (8.7) | 39 (6.5) | 38 (6.6) |
| Sedation & neuromuscular blockade | | | | | | | | | | |
| - Low dose sedation | 490 (68.8) | 451 (63.0) | | 403 (58.1) | 366 (55.1) | | | 358 (56.7) | 313 (52.4) | 288 (50.2) |
| - Higher dose sedation | 319 (44.8) | 352 (49.2) | | 328 (47.4) | 292 (44.0) | | | 253 (40.2) | 215 (36.0) | 187 (32.6) |
| - Metabolic suppression | 128 (18.0) | 148 (20.7) | | 154 (22.3) | 142 (21.5) | | | 131 (20.8) | 113 (18.9) | 103 (17.9) |
| - Neuromuscular blockade | 158 (22.2) | 147 (20.5) | | 118 (17.1) | 105 (15.8) | | | 110 (17.4) | 90 (15.1) | 81 (14.1) |
| CSF drainage |  |  | |  |  | | |  |  |  |
| - CSF drainage | 91 (12.8) | 119 (16.6) | | 123 (17.8) | 123 (18.6) | | | 134 (21.3) | 133 (22.3) | 131 (22.9) |
| CPP management |  |  | |  |  | | |  |  |  |
| - Fluid loading | 302 (42.5) | 268 (37.5) | | 211 (30.5) | 172 (26.0) | | | 142 (22.5) | 130 (21.8) | 104 (18.1) |
| - Vasopressors | 476 (66.5) | 505 (70.5) | | 460 (66.5) | 408 (61.6) | | | 354 (56.1) | 295 (49.6) | 247 (43.0) |
| Ventilatory management for ICP control | | | | | | | | | | |
| - Mild hypocapnia | 265 (37.2) | 292 (40.8) | | 268 (38.7) | 235 (35.5) | | | 211 (33.4) | 187 (31.3) | 168 (29.3) |
| - Moderate hypocapnia | 69 (9.7) | 86 (12.1) | | 68 (9.8) | 68 (10.3) | | | 58 (9.2) | 50 (8.4) | 43 (7.5) |
| - Intensive hypocapnia | 9 (1.2) | 14 (2.0) | | 15 (2.2) | 8 (1.2) | | | 11 (1.7) | 10 (1.7) | 13 (2.3) |
| Hyperosmolar therapy |  |  | |  |  | | |  |  |  |
| - Mannitol ^1^ | 62 (8.7) | 59 (8.2) | | 65 (9.4) | 48 (7.3) | | | 45 (7.1) | 46 (7.7) | 31 (5.4) |
| - Hypertonic saline ^2^ | 113 (15.9) | 119 (16.6) | | 97 (14.0) | 81 (12.2) | | | 78 (12.4) | 70 (11.7) | 70 (12.2) |
| - Mannitol high^3^ | 9 (1.2) | 14 (2.0) | | 10 (1.4) | 6 (0.9) | | | 5 (0.8) | 7 (1.2) | 10 (1.7) |
| - Hypertonic saline high ^4^ | 42 (5.9) | 47 (6.6) | | 30 (4.3) | 29 (4.4) | | | 27 (4.3) | 28 (4.7) | 26 (4.5) |
| Temperature control |  |  | |  |  | | |  |  |  |
| - Treatment of fever >38 °C | 138 (19.4) | 178 (24.8) | | 178 (25.7) | 200 (30.2) | | | 231 (36.6) | 214 (35.8) | 216 (37.6) |
| - Mild hypothermia ≥35°C | 27 (3.8) | 40 (5.6) | | 43 (6.2) | 46 (6.9) | | | 48 (7.6) | 49 (8.2) | 40 (7.0) |
| - Hypothermia <35°C | 36 (5.1) | 17 (2.4) | | 18 (2.6) | 12 (1.8) | | | 15 (2.3) | 17 (2.8) | 12 (2.1) |
| Surgery for intracranial hypertension | |  | |  |  | | |  |  |  |
| - Intracranial operation | 53 (7.5) | 22 (3.1) | | 11 (1.6) | 5 (0.8) | | | 6 (0.9) | 5 (0.8) | 2 (0.3) |
| - Decompressive craniectomy | 0 * | 11 (1.5) | | 8 (1.2) | 11 (1.7) | | | 7 (1.1) | 12 (2.0) | 8 (1.4) |
| Mean TIL score | 6.7 | | 6.9 | 6.6 | | 6.2 | 6.2 | | 6.0 | 5.7 |
| This table shows the number and percentages of ICP-monitored patients receiving ICP-lowering treatments on the TIL scale.  Each row shows the number of patients (frequency) that receive that treatment (a patient could receive multiple treatments per topic)  Low dose sedation as required for mechanical ventilation; higher dose sedation for ICP control (not aiming at burst suppression); metabolic suppression for ICP control (with high dose barbiturates or propofol); mild hypocapnia (PaCO2 4.6-5.3 kPa), moderate hypocapnia (PaCO2 4.0-4.5 kPa), intensive hypocapnia (PaCO2 < 4.0 kPa); intracranial operation for progressive mass lesion not scheduled on admission). Percentages take missing values into account  * Decompressive craniectomies were excluded from day 1. This represents the incidence per day (in our analyses this is the prevalence)    1) 2 g/kg/24 hours, 2) 0.3 g/kg/24hours, 3) > 2 g/kg/24 hours, 4) > 0.3 g/kg/24 hours  ICU: intensive care unit, TIL: therapy intensity level | | | | | | | | | | |
