# Supplementary material for: Use and impact of high intensity treatments in patients with traumatic brain injury across Europe: a CENTER-TBI analysis
Source: Crit Care. 2021 Feb 23;25:78. doi: 10.1186/s13054-020-03370-y (PMC7901510; doi:10.1186/s13054-020-03370-y)
Supplement: Supplementary file 4 — Additional file 4. Daily TIL scores. Description: This figure shows the daily high TIL scores (cumulative score of the high TIL treatments) plotted against the daily low TIL scores (cumulative score for low TIL treatments). It shows that at the same high TIL scores a variety of low TIL treatment (scores) is applied (in some cases even no low TIL treatment). Also, the figure shows mainly at day 3 (dark green) higher TIL treatments are applied including higher low TIL scores. [file 13054_2020_3370_MOESM4_ESM.docx]

Supplement 4


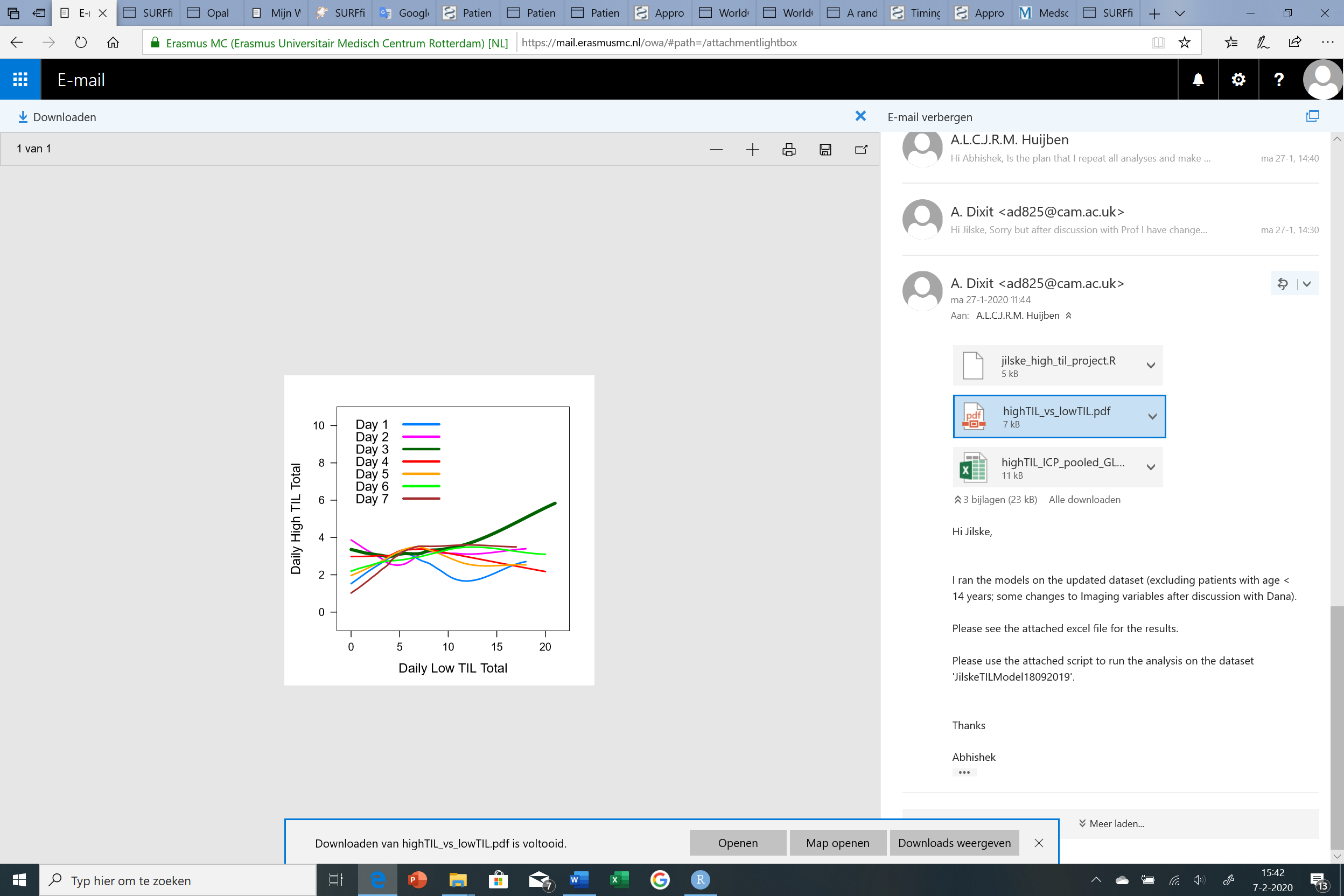


This figure shows the daily high TIL scores (cumulative score of the high TIL treatments) plotted against the daily low TIL scores (cumulative score for low TIL treatments). It shows that at the same high TIL scores a variety of low TIL treatment (scores) is applied (in some cases even no low TIL treatment). Also, the figure shows mainly at day 3 (dark green) higher TIL treatments are applied including higher low TIL scores.

TIL: therapy intensity level
