# Supplementary material for: Use and impact of high intensity treatments in patients with traumatic brain injury across Europe: a CENTER-TBI analysis
Source: Crit Care. 2021 Feb 23;25:78. doi: 10.1186/s13054-020-03370-y (PMC7901510; doi:10.1186/s13054-020-03370-y)
Supplement: Supplementary file 6 — Additional file 6. Higher tier ICP-lowering treatments in patients receiving high TIL treatment. Description: This figure shows the proportion of patients that receive first and second tier treatments of the high TIL patients across 7 days at the Intensive Care Unit. [file 13054_2020_3370_MOESM6_ESM.docx]

Supplement 6


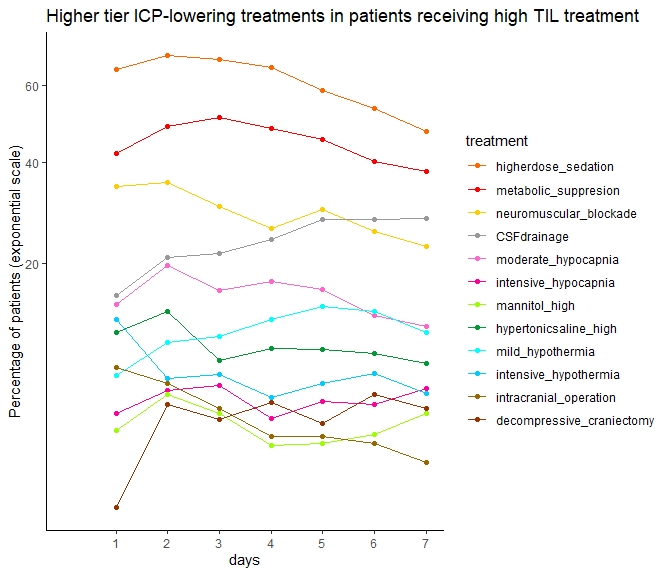


This figure shows the proportion of patients that receive first and second tier treatments of the high TIL patients across 7 days at the Intensive Care Unit. Decompressive craniectiomies at day 1 were excluded. Mannitol_high: >2 g/kg/24h, hypertonicsaline_high: > 0.3 g/kg/24h
