# Supplementary material for: Use and impact of high intensity treatments in patients with traumatic brain injury across Europe: a CENTER-TBI analysis
Source: Crit Care. 2021 Feb 23;25:78. doi: 10.1186/s13054-020-03370-y (PMC7901510; doi:10.1186/s13054-020-03370-y)
Supplement: Supplementary file 7 — Additional file 7. Variation in high TIL treatment percentages across centres (centre-level). Description: This table describes the between-centre variation for high TIL treatments across the days. The number of centres are the centres that actually apply the individual treatments. The mean percentage represents the mean percentage of patients across centres that receive the therapy, while the IQR and min-max represents the variation in treatment use between centres. [file 13054_2020_3370_MOESM7_ESM.docx]

Additional file 7. Variation in high TIL treatment percentages across centres (centre-level)

| Table 4. Percentages of high TIL treatment across centres | | | |  |  |  |  |  |
| --- | --- | --- | --- | --- | --- | --- | --- | --- |
| Treatment use | Number of centres  (N, %) | Day 1 | Day 2 | Day 3 | Day 4 | Day 5 | Day 6 | Day 7 |
| 1. Barbiturates   Mean %  IQR  Min-max | 46 (88.5) | 18  0-27  0-100 | 22  0-36  0-80 | 25  0-40  0-100 | 22  0-35  0-100 | 19  0-28  0-78 | 17  0-24  0-75 | 17  0-25  0-100 |
| 1. Hypothermia below 35 °C   Mean %  IQR  Min-max | 32 (61.5) | 5  0-6  0-50 | 4  0-0  0-100 | 2  0-0  0-20 | 1  0-0  0-14 | 2  0-0  0-33 | 2  0-0  0-25 | 1  0-0  0-14 |
| 1. Intensive hyperventilation   Mean %  IQR  Min-max | 21 (40.4) | 2  0-0  0-50 | 3  0-0  0-50 | 4  0-0  0-100 | 1  0-0  0-33 | 1  0-0  0-14 | 1  0-0  0-25 | 1  0-0  0-25 |
| 1. Decompressive craniectomy   Mean %  IQR  Min-max | 26 (50.0) | * | 2  0-0  0-50 | 2  0-0  0-20 | 1  0-0  0-17 | 1  0-0  0-17 | 1  0-0 0-17 | 1  0-0  0-17 |
| Overall high TIL use  Mean %  IQR  Min-max | 47 (90.4) | 42  25-51  0-100 | 43  27-52  0-100 | 43  27-56  0-100 | 43  27-56  0-100 | 43  27-55  0-100 | 42  26-57  0-100 | 43  27-59  0-100 |
| This table describes the between-centre variation for high TIL treatments across the days. The number of centres are the centres that actually apply the individual treatments. The mean percentage represents the mean percentage of patients across centres that receive the therapy, while the IQR and min-max represents the variation in treatment use between centres.  * We excluded patients with decompressive craniectomy at day 1 as we considered this a different treatment group  IQR: interquartile range, min: minimum, max: maximum, TIL: therapy intensity level | | | | | | | | |
