# Supplementary material for: Use and impact of high intensity treatments in patients with traumatic brain injury across Europe: a CENTER-TBI analysis
Source: Crit Care. 2021 Feb 23;25:78. doi: 10.1186/s13054-020-03370-y (PMC7901510; doi:10.1186/s13054-020-03370-y)
Supplement: Supplementary file 8 — Additional file 8. Baseline characteristics matched dataset. Description: This table describes the baseline characteristics of the matched cases with complete data (as the dataset was imputed, this table could only be completed for complete cases). Significant group differences were determined by using the chi-square or Fisher’s exact test (non-normal distributions) for categorical variables and an ANOVA or Kruskal Wallis test (non-normal distributions) for continuous variables. [file 13054_2020_3370_MOESM8_ESM.docx]

Additional file 8. Baseline characteristics matched dataset

| Table 5. Baseline patient characteristics matched dataset (complete cases) | | | | |
| --- | --- | --- | --- | --- |
|  | Low TIL  (*N*= 152) | High TIL  (*N*= 127) | *p*-value | Standardized mean difference |
| IMPACT variables |  |  |  |  |
| Age (median, IQR)  GCS motor score (N, %)  1  2  3  4  5  6  GCS pupils (N, %)  0  1  2  Marshall (N, %)  1  2  3  4  5/6  Hypoxia (N, %)  Hypotension (N, %)  Hemoglobin (median, IQR)  Glucose (median, IQR) | 48 [27.0- 62.2]  68 (44.7)  7 (4.6)  7 (4.6)  10 (6.6)  36 (23.7)  24 (15.8)  116 (76.3)  13 (8.6)  23 (15.1)  2 (1.3)  74 (48.7)  14 (9.2)  5 (3.3)  57 (37.5)  21 (13.8)  19 (12.5)  13.2 [11.6- 14.4]  8.1 [6.8- 9.8] | 42.0 [26- 58.5]  64 (50.4)  9 (7.1)  7 (5.5)  14 (11.0)  20 (15.7)  13 (10.2)  98 (77.2)  12 (9.4)  17 (13.4)  1 (0.8)  43 (33.9)  32 (25.2)  4 (3.1)  47 (37.0)  18 (14.2)  17 (13.4)  13.1 [11.8- 14.2]  7.8 [6.7- 9.6] | 0.361  0.244  0.898  0.006  1.000  0.968  0.769  0.494 | 0.103  0.015  0.110 |
| CT variables |  |  |  |  |
| EDH (N, %)  tSAH (N, %)  Brain herniation (N, %)  Cortical sulcus effacement (N, %)  Ventricular compression(N, %)  Midline shift(N, %) | 35 (23.0)  128 (84.2)  32 (21.1)  38 (25.0)  52 (34.2)  42 (27.) | 27 (21.3)  117 (92.1)  28 (22.0)  31 (24.4)  52 (40.9)  32 (25.2) | 0.835  0.067  0.956  1.000  0.301  0.747 |  |
| Additional variables |  |  |  |  |
| ISS without head injury (median, IQR  Max ICP ^a^ (median, IQR) | 13 [1-25]  22 [16-31] | 9 [0-25]  22 [14.5-26] | 0.645  0.105 | 0.020  0.149 |
| This table describes the baseline characteristics of the matched cases with complete data (as the dataset was imputed, this table could only be completed for complete cases). Significant group differences were determined by using the chi-square or Fisher’s exact test (non-normal distributions) for categorical variables and an ANOVA or Kruskal Wallis test (non-normal distributions) for continuous variables.   1. max ICP prior to treatment   GCS: Glasgow Coma Scale, IMPACT: International mission for prognosis and analysis of clinical trials in TBI, ISS: injury severity score, IQR: interquartile range, TIL: therapy intensity level | | | | |
