# Supplementary material for: Use and impact of high intensity treatments in patients with traumatic brain injury across Europe: a CENTER-TBI analysis
Source: Crit Care. 2021 Feb 23;25:78. doi: 10.1186/s13054-020-03370-y (PMC7901510; doi:10.1186/s13054-020-03370-y)
Supplement: Supplementary file 9 — Additional file 9. Maximum ICP values prior to start high TIL use per treatment group. Description: This figure shows the differences in maximum ICP values prior to high TIL treatment between patients with a high TIL (1) versus a low TIL treatment (0). The median ICP for low TIL is 22 [16-28] and for high TIL 22 [16-27]. This difference is not statistically significant. [file 13054_2020_3370_MOESM9_ESM.docx]

Additional file 9. Maximum ICP values prior to start high TIL use per treatment group


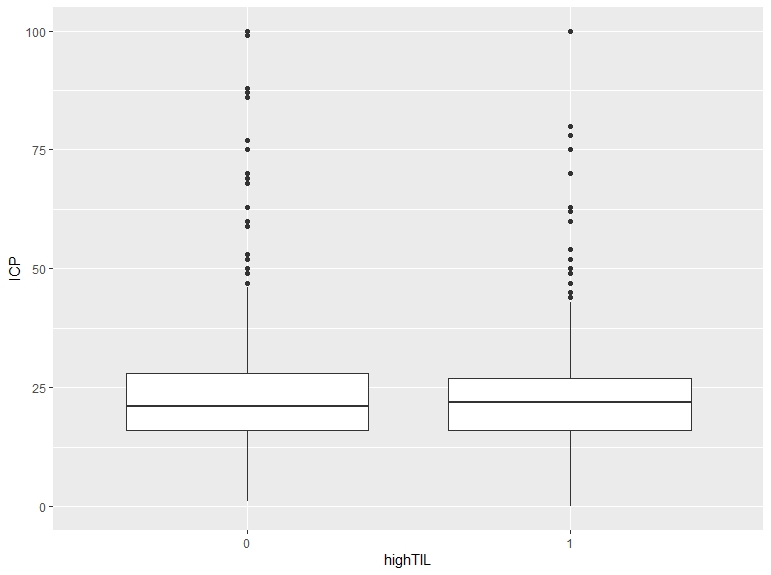


This figure shows the differences in maximum ICP values prior to high TIL treatment between patients with a high TIL (1) versus a low TIL treatment (0). The median ICP for low TIL is 22 [16-28] and for high TIL 22 [16-27]. This difference is not statistically significant.
